# Early predictors of acute hepatitis B progression to liver failure

**DOI:** 10.1371/journal.pone.0201049

**Published:** 2018-07-26

**Authors:** Qing-Fang Xiong, Tian Xiong, Ping Huang, Yan-Dan Zhong, Hua-Li Wang, Yong-Feng Yang

**Affiliations:** 1 Liver Disease Department, The Second Hospital of Nanjing, affiliated to Medical School of South-East University, China; 2 Department of Anesthesiology, Beijing Aerospace General Hospital, Beijing 100076, China; Centre de Recherche en Cancerologie de Lyon, FRANCE

## Abstract

**Background and aims:**

1~4% of acute hepatitis B (AHB) cases in adults progresses to acute liver failure (ALF).The predictors of ALF and prognosis for patients with ALF are not clear. This study investigated some of predictive and prognostic factors for AHB progression to ALF.

**Methods:**

A retrospective analysis was used to assess the clinical and laboratory features of 293 patients diagnosed with AHB; the patients were divided into the following two groups: ALF (n = 13) and non-ALF (n = 280).

**Results:**

In total,13 of the 293 (4.43%) patients developed ALF (10 recovered、3 died). The variables of age, anti-HBc IgM titers≥10 S/CO, HBeAg negativity, and total bilirubin (TB) at admission were significantly higher in ALF patients than in non-ALF patients. Compared to non-ALF patients, ALF patients had significantly lower values for prothrombin time activity (PTA), serum albumin, and HBV DNA. At discharge, ALF patients had lower TB normalization rates and much faster clearance of HBsAg, HBeAg and HBVDNA than non-ALF patients. In multivariate analysis, TB≥5×upper limit of normal (ULN) and HBeAg negative status were independent predictors for ALF development at admission, with 84.6% sensitivity, 85.7% specificity, a likelihood ratio of 5.91 and an area under the receiver operating characteristics curve (AUROC) of 0.850.Those who died had lower levels of peak PTA (<20%) and higher levels of peak hepatic encephalopathy (HE) grade (III-IV) than those who recovered.

**Conclusions:**

Of the patients with ALF, 23.1% died. TB≥5×ULN and HBeAg negative status were the most effective and practicable factors distinguishing ALF from AHB at admission before the onset of encephalopathy. Peak PTA<20% and/or HE grade III-IV were independent predictors of a high probability of death or a need for transplantation.

## Introduction

China has a high prevalence of HBV infection. The number of newly infected patients has significantly declined due to the universal vaccination implemented in newborns whose mothers are carriers; however, there are still some adult patients who have not been vaccinated or in whom the vaccination has failed who could still be infected by sexual exposure or intravenous drug use. AHB in adults can lead to a wide spectrum of liver disease ranging from asymptomatic and inapparent to acute symptomatic hepatitis, ALF and fatal acute infections (fulminant). On the other hand,90% of patients recover from AHB, while 10% develop chronic hepatitis B (CHB),1~4% of severe acute hepatitis B cases can rapidly progress to ALF [1]. Approximately 20~80% of ALF cases result in death or transplantation [2, 3]. Thus, it is important to distinguish severe AHB patients who are likely to progress to ALF at an early stage and ALF patients who require liver transplantation from those patients who will survive with comprehensive medical treatment.

It has been reported that older individuals [4,5],jaundice[6],decreased hepatitis B e-antigen (HBeAg) expression[7], viral mutations [pre-core (G1896A, G1899A), core promoter (T1753A/C, T1754C/G, A1762T/G1764A)] [6],alcohol and methamphetamine abuse[7], and genotype D [8] are risk factors for the development of severe disease. However, these results were based on data only from AHB-related ALF (AHB-ALF) patients and CHB-related ALF patients, or including patients with ALF caused by other factors (hepatitis A or E or even less frequently CMV, HSV, VZV, and dengue) [9,10].

Various prognostic indicators and scoring systems have been used to predict outcomes in ALF, including the Child-Pugh score and the model for end-stage liver disease score (MELD).The Child-Pugh score is a reasonably reliable predictor of survival for patients with cirrhosis and provides the standard criteria for listing for liver transplantation (Child-Pugh class B) [11, 12]. The MELD is calculated using TB, serum creatinine, and PT-INR, and provides a more objective method of assessing disease severity than the Child-Pugh score; the MELD is currently used to establish the priority listing for liver transplantation [11, 12]; however, renal failure is a terminal events in ALF, and it is difficult to find a donor liver in such a short time. Hence, in this study, we performed an extensive investigation with 293 hospitalized adult AHB patients admitted to our department from 2010 to 2015, in order to compare the clinical and laboratory changes in acute hepatitis B with and without liver failure, to predict the progression of acute hepatitis B to liver failure at admission before the onset of encephalopathy, and to investigate the prognostic markers of ALF in a population in Nanjing, China.

## Patients and methods

### Patients and diagnosis

We collected and analyzed the clinical and laboratory data of hospitalized adult patients with AHB confirmed by laboratory tests in the Second Hospital of Nanjing (the hospital in Nanjing that is in charge of the diagnosis and treatment of the viral hepatitis) between January 1, 2010 and December 31, 2015. This was a retrospective observational noninterventional study, and reported data were analyzed anonymously. All participants provided verbal informed consent after the study had been fully explained and agreed to the interview or telephone call being audio-recorded. The study protocol was approved by the Medical Ethics Committee of the Second Hospital of Nanjing (2018-LY-kt001).

The diagnosis of AHB was based on discrete symptoms (such as fever, loss of appetite, fatigue, and dark urine),signs(jaundice), laboratory examinations[elevated serum alanine aminotransferase (ALT) or aspartate aminotransferase (AST) levels], detection of either serum IgM antibody against hepatitis B core antigen (anti-HBc IgM) or hepatitis B surface antigen (HBsAg) or both, no detection of HBsAg for 6 months before presentation, and exclusion of other possible causes of acute hepatitis (i.e., viruses, toxins, alcohol, autoimmunity, and metabolic factors) [1,2].

AHB-ALF was defined as a severe clinical syndrome with prothrombin time activity (PTA)≤40% (of normal%) and encephalopathy (any degree of altered mentation, range I~IV) in patients with AHB [13]. CHB was classified as AHB in which serum HBsAg persisted for≥ 1 year [1].All patients were followed for 6~12 months. HBV markers were checked at least monthly during the first 6 months after the onset of illness and bimonthly for the next 6 months.

### Serum assay methodology

Routine biochemical tests were performed using standardized laboratory procedures. HBsAg,the antibody against HBsAg (anti-HBs), HBeAg, and the antibody against HBeAg (anti-HBe) were measured using a microparticle enzyme immunoassay (Abbott Laboratories, North Chicago, IL, United States). Serum HBV DNA levels were measured by the VERANT 3.0 assay(Bayer Healthcare, Tarrytown, NY, United States; lower limit of detection 500 IU/mL) or COBAS TaqMan PCR assay (Roche, Branchburg, NJ, United States; lower limit of detection 20 IU/mL). IgM anti-HBc (index value, 1.0) levels were determined using the chemiluminescent immunoassay on the Abbott Architect (Abbott GmbH, Wiesbaden, Germany).

### Statistical analysis

Quantitative data were described using medians and ranges. Continuous variables were compared by Student’s t-test or nonparametric tests, while categorical variables were compared by chi-squared tests or Fisher's exact test. The logistic regression analysis model was used to estimate the univariate and multivariate effects of the different risk factors on the development of liver failure. Odds ratios (ORs) and 95% confidence intervals (CIs) were used to describe the strength of the association between the risk factors and ALF. Receiver operating characteristic (ROC) curves and likelihood ratios were calculated for the most relevant parameters. *P* value <0.05 was considered statistically significant. The results were analyzed using SPSS software version 15.0 for Windows.

## Results

### Demographic characteristics of AHB in the ALF and non-ALF groups

In total,293 hospitalized patients with AHB confirmed by laboratory tests with ages ranging from 18 to73 years (y) were enrolled, 278 (94.8%) recovered from AHB, 2 (0.83%) developed CHB, 13 (4.43%) progressed to ALF [10 (76.9%) recovered,3 (23.1%) died,0 received a liver transplant]. Four of the 13 patients who progressed to ALF were diagnosed with ALF at admission, while other patients progressed to ALF during hospitalization (5 patients had PTA≤40% without encephalopathy, 4 patients had neither PTA≤40% nor encephalopathy at admission).

The median duration of hospitalization and median time from symptom onset to hospital admission were similar between the non-ALF group and the ALF group (Table 1). The median age of the ALF patients was much greater than that of the non-ALF patients (Table 1).Patients aged 18~49 years were 4 times(224/56) more likely to be affected than those aged≥50 years in the non-ALF group, while in contrast, in the ALF group, patients older than 50 years were 1.6 times (8/5) more likely to acquire the disease than those in the 18~49 year age range; there was a significant difference in age between the ALF group and the non-ALF group[X^2^ = 12.5, *P* = 0.0003,OR = 6.4,95% CI (2.01~20.31)] (Table 2).

**Table 1:** Demographic and baseline characteristics between patients with AHB with ALF and those without ALF.

Group	N	Age(y)	Gender (M/F,N)	Time from symptom onset to hospital admission (d)	Duration of hospitalization (d)	Type 2 diabetes mellitus(N, %)	AFP(N, %)
Non-ALF	280	36(18~73)	202/78	7(2~65)	29(7~91)	13/280(4.64)	7/280(2.5)
ALF	13	55(22~67)	11/2	8(5~60)	30(5~75)	1/13 (7.69)	3/13(23.1)
*P*		0.047	0.324	0.112	0.484	0.61	<0.001

NOTE. Age, time from symptom onset to hospital admission and duration of hospitalization are expressed as medians (and ranges), others data are presented as number and percent. ALF: acute liver failure; AFP: alpha fetoprotein; M: male; F: female; N: number; d: day; y: years.

**Table 2:** Multivariate analysis for factors independently associated with ALF in AHB.

Factors	Univariate OR (95% CI)	*P* value	Multivariate OR (95% CI)	*P* value
TB (μmol/L) <5×ULN vs. ≥5×ULN	17.73(2.27~138.30)	<0.001	8.07 (0.89~72.64)	0.033
HBeAg positive vs.negative	8.27(1.79~38.06)	0.001	6.49(1.14~36.96)	0.035
Age(y) ≥50 vs.<50	6.4(2.01~20.31)	0.0003	1.34(0.25~7.17)	0.731
anti-HBc IgM (S/CO) ≥10 vs.<10	0.22(0.04~1.02)	0.035	0.17(0.01~1.54)	0.115
HBVDNA (log10 IU/mL) ≥5.0 vs. <5.0	0.95(0.92~0.98)	0.163	0 (0.0)	0.997

NOTE. ALF: acute liver failure; AHB: acute hepatitis B; TB: total serum bilirubin; anti-HBc IgM: serum IgM antibody against hepatitis B core antigen; HBeAg: hepatitis B e antigen.

**Fig 1:**
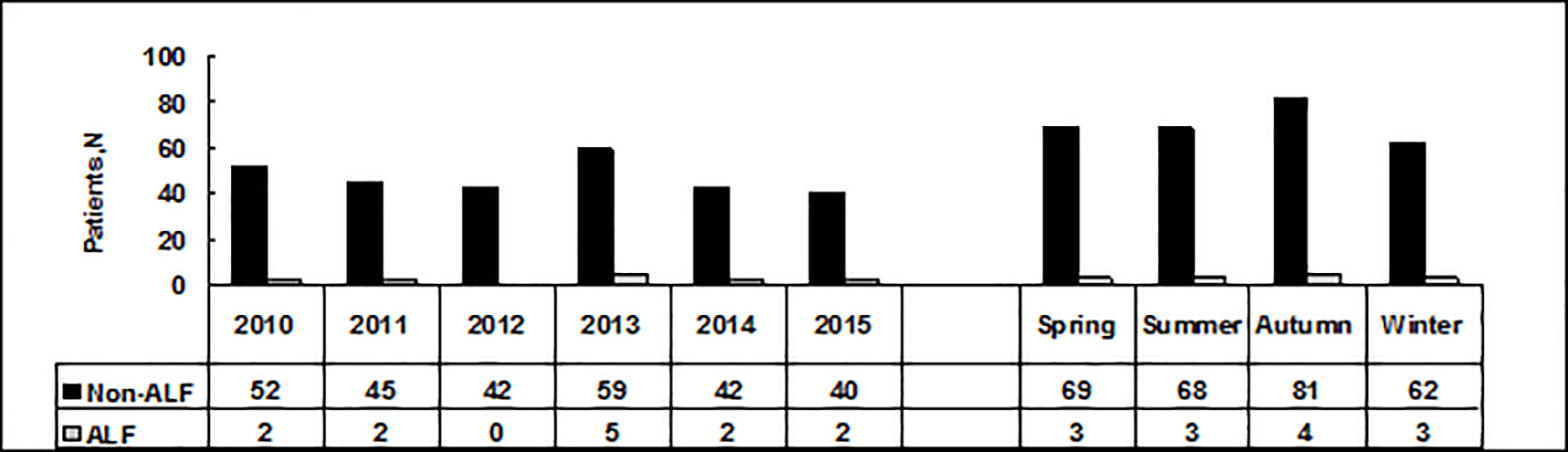
The seasonality and annual numbers of adult patients with AHB with and without ALF from 2010 to 2015. Annual and seasonal comparisons had X2 = 3.75, P = 0.585 and X2 = 0.03, P = 0.998 respectively. ALF: acute liver failure.

There were no differences in the ratio of males to females,the prevalence of type 2 diabetes mellitus (Table 1),or the seasonality and annual numbers of adult patients from 2010 to 2015 between the two groups(X^2^ = 0.03,*P* = 0.998; X^2^ = 3.75,*P* = 0.585,respectively) (Fig 1).

### Biochemical characteristics of AHB in the ALF and non-ALF groups

At admission, jaundice was more frequently observed in the ALF group than in the non-ALF group[13/13(100%) vs. 215/280 (76.8%); X^2^ = 3.87, *P* = 0.049).The median total bilirubin (TB) levels of ALF patients were significantly higher than those of the non-ALF patients (Table 3).The ALF group had TB≥5×ULN [12/13(92.3%) vs.113/280(40.4%), X^2^ = 13.7, *P*<0.001; OR = 17.73; 95%CI (2.27~138.30)] (Table 2); the median ALT and AST values were similar in the two groups (Table 3). Median serum albumin levels were significantly lower in the ALF group than in the non-ALF group (Table 3).The median PTA was lower in the ALF group than in the non-ALF group [n = 13, 44.4%(0~80.7)vs.n = 276, 78.1%(35.2~144.6); *P*<0.001] (Table 4).The AFP levels were significantly elevated in ALF patients compared to those of non-ALF patients (Table 1).

**Table 3:** Biochemistry characteristics between patients with AHB with ALF and without ALF at admission and at the peak.

		TB(μmol/L)		ALT(IU/L)		AST(IU/L)		Serum albumin(g/L)	
Group	N	At admission	Peak	At admission	Peak	At admission	Peak	At admission	Peak
Non-ALF	280	76.1(4.8~309.4)	78.95(4.8~469.3)	1143.6(36.6~4756.5)	963.7(36.6~4448.3)	434.95(14~6721)	369.5(14~3115.8)	40.5 (27.9~47.2)	42.4(37~47.2)
ALF	13	180.2(90.1~382.4)	254.8(90.1~545.6)	1085.3(130.2~3974.5)	356.4(49.3~3974.5)	547.0(100~3341.2)	105.2(42.8~1684.4)	32.1(25~37)	33.6(25~40.4)
*P*		<0.001	<0.001	0.70	0.047	0.085	0.073	<0.001	<0.001

NOTE. All variables are expressed as medians (and ranges). ALF: acute liver failure; TB: total serum bilirubin; ALT: alanine aminotransferase; AST: aspartate aminotransferase. N: number

**Table 4:** Serum tests between patients with AHB with ALF and without ALF.

Group	N	Inconsistence of TB with peak ALT levels (N, %)	PTA(Of normal%)	HBVDNA(log10 IU/mL)	Anti-HBcIgM S/CO≥10(N,%)	HBeAg negative(N,%)
Non-ALF	280	41/280(14.64)	78.1(35.2~144.6)	3.97(2.2~7.41)	154/280(55.0)	109/273(39.9)
ALF	13	8/13(61.53)	44.4(0~80.7)	2.98(2.72~4.10)	11/13 (84.6)	11/13(84.6)
*P*		<0.001	<0.001	0.011	0.035	0.001

NOTE. Peak PTA and HBVDNA are expressed as medians (and ranges); the others are stated as number and percent. ALF: acute liver failure; PTA: prothrombin time activity; TB: total serum bilirubin;ALT: alanine aminotransferase; anti-HBc IgM: serum IgM antibody against hepatitis B core antigen; HBeAg: hepatitis B e-antigen. N: number; S/CO: sample/cutoff.

At the peak stage, the inconsistence of TB with peak ALT levels (enzyme-jaundice separation) was greater in ALF than in non-ALF patients (Table 4), and the median TB of ALF patients was significantly higher than that of non-ALF patients (Table 3).The median ALT and serum albumin levels were lower in the ALF group than in the non-ALF group (Table 3). The median AST was not different between two groups (Table 3).

At discharge, the clinical manifestations and biochemical data of all patients had significantly improved (excluding the 3 deaths). The TB normalization rates were higher in non-ALF patients than in ALF patients [246/280(87.9%) vs.5/10(50%); X^2^ = 11.88; *P* = 0.001].ALT normalization rates were similar in the two groups [236/280(84.3%) vs. 8/10(80%); X^2^ = 1.33, *P* = 0.715].After 6 (5.2–8.3) months [mean (min-max)] of follow-up, ALT and TB normalization rates were similar in the two groups (100%).

### HBV virological characteristics of AHB in the ALF and non-ALF groups

All patients were IgM anti-HBc positive at admission.The ALF group had higher IgM anti-HBc titers (≥10 S/CO) than the non-ALF group [11/13 (84.6%) vs. 154/280 (55.0%), X^2^ = 4.42, *P* = 0.035)] (Table 4). The distribution of HBV genotypes B, C and others were similar in the ALF group [7/13(53.8%) vs. 6/13(46.2%) vs.0%] and in the non-ALF group [146/280(52.1%) vs.126/280(45%) vs.8/280(2.9%), X^2^ = 0.382, *P* = 0.826).

At admission, the HBsAg>250 IU/mL proportion [147/273(53.8%) vs. 3/13(23.1%); X^2^ = 4.77, *P* = 0.029] and HBeAg positive proportion [164/273(60%) vs.2/13(15.4%); X^2^ = 10.17, *P* = 0.001] were greater in the non-ALF group than in the ALF group (Tables 2 and 4). In the non-ALF group, the HBeAg positive and anti-HBe positive accounted for 60.0% (164/273) and 22.3% (61/273), respectively. In the ALF group, 2 (15.4%) patients were HBeAg positive,3 (23.1%) patients were anti-HBe positive,and 4(30.7%) patients were both HBsAg and anti-HBs positive,and 2(15.4%) patients were HBsAg negative. The median HBV DNA level[n = 205, 3.97(2.2~7.41)vs.n = 7, 2.98(2.72~4.10) log10 IU/mL,*P* = 0.011] was higher in the non-ALF group than in the ALF group (Table 4).

At discharge, all ALF patients had undetectable levels of HBsAg(excluding those who died). There were more patients with HBsAg serological loss in the ALF group than in the non-ALF group [n = 10, 10/10(100%) vs.n = 266,136/266(51.1%); X^2^ = 9.23, *P* = 0.002]. In the non-ALF group, the most common HBV marker was anti-HBe(111/266, 41.7%),while only 5.3%(14/266) was HBeAg positive. At the final follow-up, all patients had HBsAg serological conversion in the ALF group. Two patients of the 280(0.71%) persisted as HBsAg positive in the non-ALF group.

### Predictive and prognostic factors associated with ALF following AHB

Logistic regression analysis considering age (≥50 years),TB (≥5×ULN), HBeAg negative status,IgM anti-HBc levels(≥10 S/CO),and HBVDNA levels(<5.0 log10 IU/mL) at admission was employed to determine independent predictors of AHB-ALF.TB≥5×ULN and HBeAg negative status were independently associated with AHB progression to ALF (Table 2).

**Fig 2:**
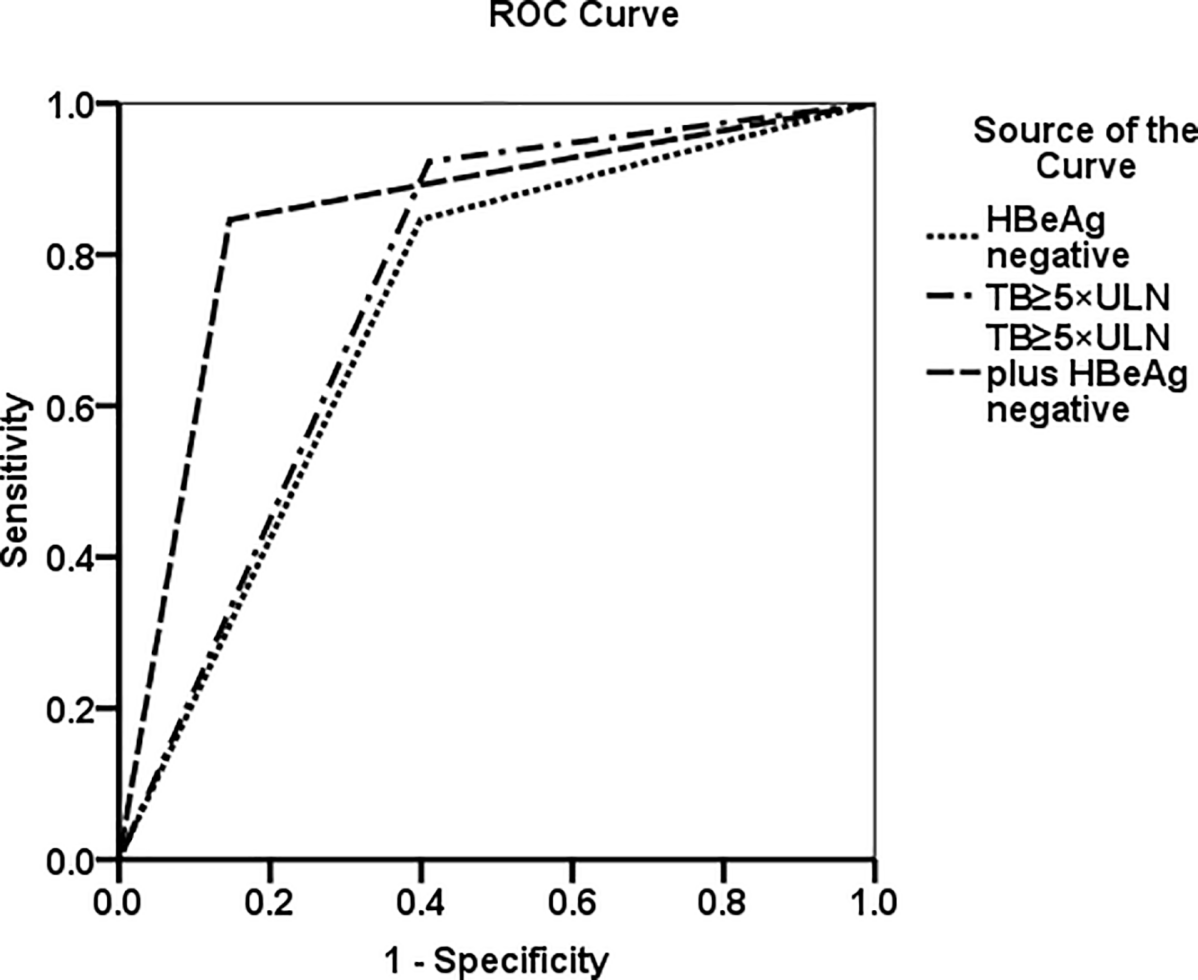
The prediction of ALF progression was analyzed with a receiver operating characteristic curve by using TB≥5×ULN, HBeAg negative and TB≥5×ULN plus HBeAg negative.The AUROC values were 0.756, 0.723 and 0.850, respectively. AUROC: area under the receiver operating characteristic curve; ALF: acute liver failure; TB: total serum bilirubin; HBeAg: hepatitis B e-antigen. ULN: the upper limit of normal.

TB≥5×UNL had 92.3% sensitivity, 59.2% specificity,a likelihood ratio of 2.26 and an area under the ROCs curves (AUROC) of 0.756 for diagnosing ALF. In addition,HBeAg negative status presented 84.6% sensitivity,60.1% specificity,a 2.12 likelihood ratio and an AUROC of 0.723 for diagnosing ALF. Combining the tests provided a diagnostic sensitivity of 84.6%, a specificity of 85.7%, a likelihood ratio of 5.91 and an AUROC of 0.850 for diagnosing ALF (Table 5, Fig 2).

**Table 5:** Comparison of screening tests for ALF in AHB patients.

Factors	Sensitivity %	Specificity %	Likelihood Ratio
TB≥5×ULN	92.3	59.2	2.26
HBeAg negative	84.6	60.1	2.12
TB ≥5×ULN + HBeAg negative	84.6	85.7	5.91

NOTE. ALF: acute liver failure; AHB: acute hepatitis B;TB:total serum bilirubin; HBeAg:hepatitis B e-antigen; ULN: the upper limit of normal.

Differences in demographics and clinical parameters were compared between those who spontaneously recovered and those with a poor outcome (death, no transplantation) in ALF. Ten of the 13(76.9%) patients survived. Those who died had a lower levels of peak PTA (<20%) [2/3(66.6%) vs.1/10(10%), X^2^ = 4.17, *P* = 0.041] and higher hepatic encephalopathy grade (Ⅲ ~ Ⅳ) [3/3(100%) vs.2/10(20%), X^2^ = 6.24, *P* = 0.012] than those who spontaneously recovered. Age, biochemistry, and virological factors were not different between the two groups. One patient died of hepatorenal syndrome, and two died of multiple organ failure syndromes (MOFS, systemic inflammatory response syndrome and hepatorenal syndrome).No patients received liver transplantation.

## Discussion

ALF is a rare but serious clinical syndrome characterized by sudden loss of hepatic function in a person without evidence of preexisting liver disease. Patients with ALF have large differences in outcomes according to etiology and geographical region [14–16]. The common causes of ALF are drug toxicity, indeterminate etiology and acute viral hepatitis [15–17]. The overall mortality of ALF without liver transplantation remains 20%-40% [18].The major reasons that lead to rapid disease progression and the highly unpredictable outcomes are not well understood.Therefore, it is difficult to take appropriate measures early to prevent poor prognosis (death) [13].

In this study, the annual number of adulthood AHB and ALF cases remained stable from January 2010 to December 2015, which may suggest that HBV preventive measures are even more rigorous for adults; it is important to publicize and improve vaccination in adults, which can seriously reduce the incidence of AHB [19, 20]. The rates of overall survival in patients with acute hepatitis B virus infection were 98.9%. However, ALF occurred in 4.43% patients with acute disease and had a mortality rate of up to 23%; the ALF occurrence rate in our patients was higher than those reported in other countries and in other areas of China(approximately 1%)[17,21], which can be explained by the fact that this study only analyzed AHB in the hospital setting. The median age of ALF patients was much older than that of non-ALF patients, and higher rates of ALF were observed in patients≥50years, however, 80% of acute HBV infections (non-ALF) were in patients younger than 50 years.Our result was similar to that in many other reports wherein older age has been associated with the severity of various viral liver diseases, with a significant difference in prognosis [4,5].

Jaundice is the first symptom in ALF [13]. Jaundice has been reported to develop in approximately 14%~30% of infected individuals. TB level usually correlates positively with the severity of liver injury in acute hepatitis [22]. In this study, hyperbilirubinemia was significantly more frequent and more severe in patients with ALF. In total, 92.3% of TB was≥5×ULN in ALF patients; it was a key predictor of ALF development. This finding was similar to those in other reports on ALF (excluding paracetamol-induced ALF, for which hyperbilirubinemia is not a prognostic factor.) [23].We observed that TB continued to increase despite decreasing serum aminotransferase levels; this phenomenon of “enzyme-jaundice separation” was more severe in patients with ALF, which was also a sensitive indicator of liver injury [24, 25]. At discharge, the normalization rates of TB and ALT were not 100%; however, their levels approached the normal range, indicating that recovery was good and quick in acute hepatitis B virus infection in adults.

In this study, patients with ALF had higher IgM anti-HBc titers (≥10 S/CO) than patients without ALF. A higher IgM anti-HBc level meant a more robust immune response in ALF patients; it showed that a strong immunologic response promoted B-cell differentiation into IgM-producing plasma blasts and high titers of IgM antibody [26]. Higher IgM anti-HBc titers can accurately distinguish acute from nonacute cases [9, 10]. At admission, 46.2% (6/13) of HBV DNA and 84.6% (11/13) HBeAg had become undetectable in patients with ALF. HBeAg negative status may signify hepatic failure.HBsAg and HBV DNA levels also fell rapidly as liver failure developed, and some patients (7/13, 53.8%) were HBsAg negative by the time of onset of hepatic encephalopathy. The reason is the rapid and extensive elimination of HBV by the vigorous host immune responses [25,27].On the other hand, AHB patients without ALF eliminated HBV slowly, with 60% of patients HBeAg positive at admission; after a median (min-max) of 29(7~91) days of supportive treatment, HBeAg became undetectable or seroconverted in 91.4% (150/164) of patients, and HBVDNA became undetectable in 199/205(97.1%)(normal range<500IU/ml).However,2 patients without ALF progressed to CHB, while ALF patients didn't evolve to a chronic disease; these phenomena suggest that a strong immune response including innate immunity and antigen-specific immune responses in patients with liver failure caused more severe liver damage and more rapid virus clearance [27, 28].

PTA≤40% (of normal %) and encephalopathy are useful diagnostic markers in ALF. Patients with PTA≤40% but without encephalopathy were diagnosed as having a severe type of acute hepatitis [29]; some patients may not present with HE in the early stage, while others may have minimal encephalopathy that is difficult to recognize because it is poorly characterized.Therefore, it is difficult to distinguish between ALF and severe acute hepatitis that could likely progress to ALF at admission. In this study, TB≥5×ULN and HBeAg negative status were predictors of disease progression from AHB to liver failure at admission. The use of TB levels≥5×ULN or HBeAg negative status as an initial predictor was highly sensitive but had a low specificity for this diagnosis (Table 2, Table 5 and Fig 2). However, by combining these two markers, the sensitivity and specificity for the diagnosis of ALF were increased, and these easily measured predictive factors can quickly distinguish ALF from AHB at admission before the onset of encephalopathy [13]. It may be helpful in determining more appropriate therapeutic strategies,for example early antiviral treatment,artificial liver support (plasma exchange and hemodiafiltration), even listing for transplantation;effective prevention and treatment of complications; and improved critical care management[24,30–32]. The overall mortality of patients with ALF decreased [18]. Nucleoside or nucleotide analogues were not used during 2010 and 2012 in this retrospective study, so it was difficult to assess its role in AHB progression to ALF (data of patients who used entecavir and/or glucocorticoid steroids are shown in S1 and S2 Tables).

In this study, peak prothrombin time values of<20% of the standardized value and/or grade III or IV hepatic encephalopathy may be correlated with significantly worse outcomes (death) [33].These markers signified that these patients had transferred to a transplantation center and were being considered for liver transplantation [12]. No liver transplantation took place in this investigation; the reasons for this included the difficulties in obtaining organs in emergency situations and the economic situation of patients in China. This sample size was too small to verify these markers further.

## Conclusion

In total, 4.43% of AHB patients developed acute liver failure, and 23.1% of ALF patients died. ALF patients had more severe liver damage and much faster viral clearance.TB≥5×ULN adding to HBeAg negative status were the most effective and practicable marker for distinguishing ALF from AHB at admission.Peak PTA<20% and/or HE grade III-IV were the independent predictors of a high probability of death or the need for transplantation. One major limitation of our study was the small sample size of patients with ALF, which may have caused a type II statistical error in determining factors associated with different clinical outcomes. Further clinical and basic studies are necessary to confirm the findings.

## Supporting information

S1 Table 14 patients with severe acute hepatitis treated with entecavir and /or glucocorticoid steroids. NOTE. M: male; F: female; y: years; ALF: acute liver failure;TB: total serum bilirubin;DB:direct bilirubin; ALT: alanine aminotransferase; AST: aspartate aminotransferase;PTA: prothrombin time activity; N: number;GS: glucocorticoid steroids.
(DOC)

S2 Table Relevant data of treatment with entecavir and glucocorticoid steroids. (XLS)

S3 Table Datasets for all figures in the paper. (XLS)
