# Supplementary material for: Early predictors of acute hepatitis B progression to liver failure
Source: PLoS One. 2018 Jul 26;13(7):e0201049. doi: 10.1371/journal.pone.0201049 (PMC6062084; doi:10.1371/journal.pone.0201049)
Supplement: S1 Table — NOTE. M: male; F: female; y: years; ALF: acute liver failure;TB: total serum bilirubin;DB:direct bilirubin; ALT: alanine aminotransferase; AST: aspartate aminotransferase;PTA: prothrombin time activity; N: number;GS: glucocorticoid steroids. (DOC) [file pone.0201049.s001.doc]

| N | Age(y) | Gender(M/F) | TB(μmol/L) | DB(μmol/L) | ALT(IU/L ) | AST(IU/L ) | PTA (Of normal %) | Treatment | Diagnosis |
| --- | --- | --- | --- | --- | --- | --- | --- | --- | --- |
| 1 | 24 | M | 197.6 | 144.2 | 4756.5 | 2654.6 | 40 | GS | Non-ALF |
| 2 | 44 | M | 139.3 | 105.9 | 1195.3 | 503.3 | 35.2 | entecavir | Non-ALF |
| 3 | 46 | F | 209.4 | 127.3 | 1299.7 | 1397.6 | 39.2 | GS/entecavir | Non-ALF |
| 4 | 33 | M | 163.9 | 106.1 | 2542.5 | 883.4 | 36 | GS/entecavir | Non-ALF |
| 5 | 29 | M | 63.7 | 54.8 | 2500.5 | 1874.2 | 39.9 | GS/entecavir | Non-ALF |
| 6 | 64 | M | 184.9 | 135.2 | 1085.3 | 477.4 | 70.6 | entecavir | ALF |
| 7 | 51 | F | 110.4 | 89 | 1267.6 | 1317.3 | 80.7 | GS | ALF |
| 8 | 29 | M | 200.8 | 142.9 | 1064.3 | 173.6 | 35.8 | GS/entecavir | ALF |
| 9 | 30 | M | 231.7 | 186.5 | 274.1 | 149.7 | 30.8 | entecavir | ALF |
| 10 | 22 | F | 90.1 | 82.9 | 1977.5 | 1212 | 70.6 | GS/entecavir | ALF |
| 11 | 67 | M | 105.5 | 92.6 | 2070.7 | 2040.6 | 40 | entecavir | ALF |
| 12 | 22 | M | 169.3 | 115.6 | 3880.9 | 3341.2 | 42 | GS/entecavir | ALF |
| 13 | 57 | M | 152 | 45.6 | 1609.7 | 297.2 | 39.5 | GS/entecavir | ALF |
| 14 | 57 | M | 128 | 100 | 995 | 1000 | 39.6 | entecavir | ALF |
